# Co-occurrence of *CLCN2*-related leukoencephalopathy and SPG56

**DOI:** 10.1016/j.prdoa.2023.100189

**Published:** 2023-02-22

**Authors:** Wejdan Almasoudi, Christer Nilsson, Ulrika Kjellström, Kevin Sandeman, Andreas Puschmann

**Affiliations:** a Lund University, Skane University Hospital, Department of Clinical Sciences Lund, Neurology, Lund, Sweden; b Lund University, Skane University Hospital, Department of Clinical Sciences Lund, Ophthalmology, Lund, Sweden; c Department of Clinical Genetics and Pathology, Division of Laboratory Medicine, Office for Medical Services, Region Skåne, Sweden

**Keywords:** CYP2U1, SPG56, Hereditary spastic paraplegia, CLCN2, Leukoencephalopathy, Male infertility, Electroretinography

## Abstract

**Family Report:**

Two rare autosomal recessive neurological disorders, leukoencephalopathy with ataxia and spastic paraplegia 56 (SPG56), were found in members of the same family. Two siblings presented with spastic paraplegia, cognitive impairment, bladder and bowel dysfunction and gait ataxia; their consanguineous parents were unaffected. Ophthalmological examination revealed chorioretinopathy. Brain MRI showed T2 hyperintensities and T1 hypointensities in the internal capsules, cerebral peduncles, pyramidal tracts and middle cerebellar peduncles. Both affected siblings were homozygous for *CYP2U1* c.947A > T p.(Asp316Val), a known cause for SPG56. However, they were also homozygous for the novel variant *CLCN2* c.607G > T, p.(Gly203Cys), classified as a variant of unknown significance. Testing of additional family members revealed homozygosity for both variants in an additional brother, whom we initially considered unaffected. Both male *CLCN2* carriers were infertile, and review of the literature revealed one reported case with azoospermia, however the brother had no overt signs of SPG56. His testicular biopsy revealed incomplete maturation arrest in spermatogenesis; clinically we found mild memory impairment and hand tremor and MRI showed similar changes as his siblings. We consider *CLCN2* c.607G > T pathogenic because of the neuroradiological and clinical findings, including azoospermia.

**Conclusion:**

Considerable workup may be required to determine the pathogenicity of novel variants, and to unambiguously associate phenotype with genotype. In very rare disorders, highly specific clinical or biomarker combinations provide sufficient evidence for a variant’s pathogenicity. Phenotypic variation of monogenic disorders described in the literature may be attributed to a second co-occurring monogenic disorder, especially in consanguineous families. SPG56 may have reduced penetrance.

## Graphical abstract

**Figure:**
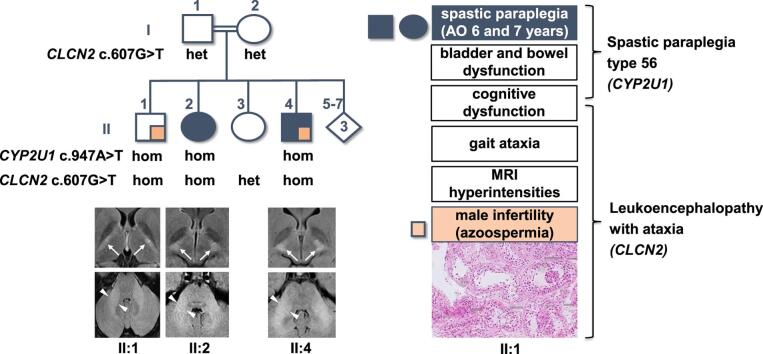

## 1 Introduction

**Fig. 1:**
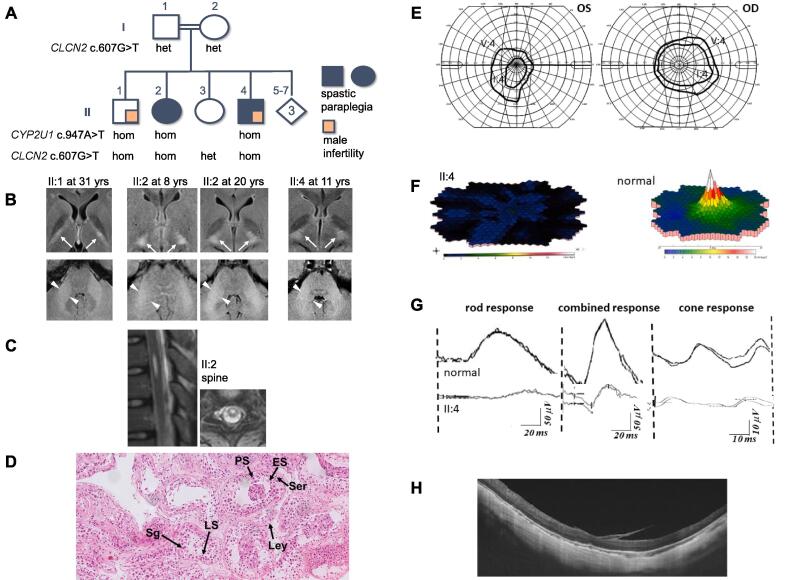
**Clinical data on the described family A** Family pedigree with genotypes: Standard symbols are used. To protect confidentiality. gender of siblings who were not examined is masked, and sibling order altered. **B** MRI images through the basal ganglia and thalamus (top) and middle cerebellar peduncles (bottom) reveal white matter hyperintensities typical for *CLCN2*-Related Leukoencephalopathy in all three homozygous carriers of the novel *CLCN2* variant. **C** MRI image of hydrosyringomyelia in the thoracal spinal cord in II:2, extending 15 mm craniocaudally. **D** Microphotograph of hematoxylin and eosin (HE) stain (20 x) of testicular biopsy from II:4. There are ample spermatogenic and primary spermatocytes. In only a few tubules, occasional secondary spermatocytes and spermatids were seen, but no obvious mature sperm cells, indicating cessation of spermatogenesis at the spermatocyte level. The average amount of mature spermatids is below 5 per tubular cross section and thus clearly lower than normal, but there was no complete lack of mature spermatids (incomplete maturation arrest). In the interstitial space spotted sloughing and degeneration with focal fibrosis was seen but a normal number of Leydig cells. Sg: spermatogonium, PS: primary spermatocyte; ES: early spermatid; LS: late spermatid; Ser: Sertoli cell; Ley: Leydig cell. **E-H** Ophthalmological examination in II:4 showed constricted visual fields (**E**) in both eyes measured by Goldmann perimetry. The central part of the visual fields could not be mapped, but multifocal electroretinography revealed severely reduced macular function (**F**) indicating that he might also have central visual field defects (scotomas) explaining the reduced visual acuity and reading difficulties. Full-field electroretinography (**G**) showed a pattern typical for cone rod degeneration with severely reduced cone amplitudes and delayed implicit times for cones, in association with a reduction of rod amplitudes. Moreover, the delayed implicit times for cones indicate a progressive disorder. Retinal structure was examined *in vivo* by optical coherence tomography (**H**) and confirmed diffuse chorioretinopathy.

Hereditary spastic paraplegia (HSP) is a group of inherited disorders characterized by progressive lower extremity weakness and spasticity. Variable additional clinical features may include intellectual disability, visual problems, ataxia and peripheral neuropathy. There are more than 70 genetic types of HSP [1]. Variability of expression has been described for HSP and many other complex neurological disorders, which can make it difficult to interpret the pathogenicity of novel variants. We describe a family with 7 children of whom two were evaluated at our clinic because of early-onset complex neurological symptomatology. Parents were consanguineous and of Iraqi origin (Fig. 1, A). Informed consent was obtained from all family members examined. The study reports examinations from clinical practice and was performed in accordance with the Declaration of Helsinki.

The first patient (**II:2**) was a 24-year-old woman born after uneventful pregnancy and normal early developmental milestones. At the age of 6 months bilateral lower extremity weakness and spasticity were noted. She became only able to walk with support but subsequently required a wheelchair. On neurological examination at age 7 years, she was alert and oriented, speech, eye movements and other cranial nerves were normal. There was leg spasticity but normal muscle tone in upper extremities. She was unable to walk. At the age of 7 brain MRI showed T_2_-weighted hyperintensities and T1-weighted hypointensities in the internal capsules, cerebral peduncles, pyramidal tracts and middle cerebellar peduncles. MRI of the spine showed mid-thoracic syringohydromyelia (Fig. 1, C). On examination at the age of 11 years, spasticity was also present in the upper limb, there were learning problems as well as urinary urgency. Nerve conduction study was normal. Regions with abnormal MRI signal (Fig. 1, B) had spread to additional areas compared to her previous MRI. By the age of 13, spastic paralysis of her right arm and hand started to develop. Repeated neuropsychological assessment from age 11 years onwards showed increasing cognitive disabilities. At the age of 11 she had mild intellectual disability, and the patient attended a school for children with special needs. At the age of 20, intellectual ability was markedly decreased. The patient was unable to perform any activities of daily living by herself. When last examined at age 24, she was wheelchair-bound, had spastic paralysis of both legs and her right arm, and showed mild ataxia in her left upper extremity. She could communicate simpler words in her second language but needed translation to her first language for longer, complex sentences. There was mild strabismus with gaze dependent diplopia of undeterminable onset, and milder myopia and visual dysfunction documented at the first contact our health service at age 9 years.

Her brother (**II:4**) was a 21-year-old man with who developed bilateral lower extremity spasticity, gait ataxia and mild cognitive problems at 6 years of age. At the age of 7 brain MRI showed changes similar to (II:2) MRI images, with white matter hyperintensities on T_2_-weighted images (Fig. 1, B). At age 13 years, his Wechsler Intelligence Scale for Children showed 46–55, indicating mild to moderate intellectual disability. At 21 years of age, he remains ambulatory, with difficulty on tandem gait and spastic gait pattern despite regular botulinum toxin injections. There was hyperreflexia in the legs with bilateral positive Babinski sign. Speech was normal but cognitive problems were apparent during conversation. There were no sensory findings. This patient had reduced vision since early childhood, certainly before 7 years of age. Ophthalmological examination including Goldmann visual fields, multifocal and full field electroretinography, and optical coherence tomography (Fig. 1, E-H) demonstrated a cone rod degeneration (chorioretinopathy) with severely reduced visual acuity, constricted visual fields and nyctalopia.

Whole exome sequencing of II:2 with filtering for homozygous variants in 3,593 genes with known association to human disease phenotypes (Blueprint Genetics, Finland) revealed a known homozygous variant for CYP2U1 NM _183075.2c.947A > T p.(Asp316Val) rs397514513 on chromosome 4q25. This variant was absent from gnomAD v3.1.1 but identified, in heterozygous carriers, in 3 of 993 exomes from the Greater Middle East Variome Project and with a variant frequency of 0.0023 in Saudi Arabian case series (Prof. Alkuraya, Riyadh, Saudi Arabia, personal communication, cited with permission). It had previously been identified in two Saudi Arabian families with autosomal recessive spastic paraplegia 56 (SPG56 – initially described as SPG49)[2], a rare early onset complex form of HSP*.* II:2′s brothers II:1 and II:4 also were homozygous for this variant. We considered many of II:2′s and II:4′s clinical features to be compatible with the core phenotype of SPG56, which includes early onset progressive lower-limb spasticity resulting in walking difficulties. The known phenotype of SPG56 also includes upper limb dystonia, cognitive dysfunction, pigmentary degenerative maculopathy, basal ganglia calcifications, thin corpus callosum and periventricular white matter hyperintensities in MRI [2], [3], [4]. Atactic symptoms have been described, albeit to our knowledge only in one individual who also was the child of consanguineous parents [5]. Some patients with SPG56 may have a subclinical sensorimotor axonal neuropathy. However, we were surprised that II:1 was a homozygous CYP2U1 mutation carrier as his clinical examination at age 34 years only revealed slight hand tremor but no additional neurological motor abnormalities; gait was normal, he was able to stand on one leg and had no spasticity and weak tendon reflexes. His family reported mild forgetfulness, but no formal cognitive testing was performed. This suggests reduced penetrance of *CYP2U1* c.947A > T.

However, whole exome sequencing had also revealed homozygosity for a previously not described variant in *CLCN2* NM_004366.5c.607G > T p.(Gly203Cys) on chromosome 3q27.1. This variant was absent from gnomAD v3.1.1, from greater than 2,500 exomes in the Greater Middle East Variome Project that were analyzed at this position, and from a Saudi Arabian NGS dataset (Prof. Alkuraya). *CLCN2* encodes for the ClC-2 plasma membrane chloride channel which plays an important role in regulating ions and water homeostasis in the brain. Homozygous or compound heterozygous mutations in *CLCN2* are known causes of Leukoencephalopathy with ataxia (*CLCN2*-related leukoencephalopathy; MIM615651), a rare autosomal recessive disease that has so far been reported in 18 individuals (Table 1). The described neurological phenotype of this disorder includes ataxia, learning disabilities, headaches, and mild visual impairment; brain MRI typically shows hyperintensities in characteristic distribution in the white matter [6]. Reviewing brain MRI images of our patients, the findings were consistent with the defined major MRI criteria, which are T2 hyperintensities in the posterior of the internal capsules, midbrain cerebral peduncles, and middle cerebellar peduncles, as well as T1-hypointensities in the same areas (Fig. 1) [6]. SPG56 also may show neuroradiological abnormalities but these are less specific and differ from the ones found in our patients, including subcortical and pallidal calcifications, periventricular white matter changes and cerebral and cerebellar atrophy [7]. However, as we only had MRI images from three family members who all were homozygous for both the *CYP2U1* and *CLCN2* variants, our data cannot provide new evidence that the MRI abnormalities seen were associated with the *CLCN2* but not the *CYP2U1* genotypes*.* We cannot exclude that some of the MRI changes might also be associated with *CYP2U1* variants; brain MRIs of only relatively few patients with SPG56 have so far been published. Nevertheless, our interpretation is that the specific and highly unusual MRI abnormalities in our patients very likely are associated with *CLCN2*-related leukoencephalopathy, and not with SPG56.

**Table 1:** Summary of all patients with *CLCN2*-related leukoencephalopathy described so far.

Individual	*CLCN2* variant	Exon	Genotype	Age at onset	Age at last examination	Clinical features	Reference
1	c.61dup p.(Leu21Profs*27) NM_004366.5	1	hom	3 m	22 m	seizures	[16]
2	c.64–1107_639del p.(Met22Leufs*5) and c.1143delT p.(Gly382Alafs*34)	2 to part of 6 and 11	comp het	12y	n.s.	mild cerebellar ataxia and a variable combination of mild spasticity, visual field defects, learning disabilities and headaches.	[17]
3 (II:2)	c.430_435del p.(Leu144_Ile145del)	4	hom	30y	n.s.	mild cerebellar ataxia and a variable combination of chorioretinopathy, psychosis	[17]
4 (II:4)	c.607G > T p.(Gly203Cys) NM_004366.5	5	hom	6 *m*	23y	combined phenotype; also has SPG56 (see main text)	present report
5 (II:1)	c.607G > T p.(Gly203Cys) NM_004366.5	5	hom	6y	21y	combined phenotype; also has SPG56 (see main text). male infertility (clinical)	present report
6	c.607G > T p.(Gly203Cys)	5	hom	n.s.	26y	male infertility (azoospermia, incomplete maturation arrest)	present report
7	c.828dupG p.(Arg277Alafs*23)	8	hom	3y	n.s.	mild cerebellar ataxia and a variable combination of mild spasticity, visual field defects, learning disabilities and headaches.	[17]
8	c.1113delinsACTGCTCAT p.(Ser375Cysfs*6)	11	hom	21y	22y	paroxysmal dyskinetic attacks	[18]
9	c.1304 T > G p.(Leu435Argfs*7)	12	hom	46y	47y	numbness, imbalance	[19]
10	c.1412G > A p.(Arg471His)	14	hom	28y	47y	gait difficulty, imbalance	[19]
11	c.1423G > A p.(Glu475Lysfs*79)	14	hom	27y	33y	headache, imbalance, blurry vision	[19]
12	c.1499C > T p.(Ala500Val)	14	hom	6y	n.s.	mild cerebellar ataxia and a variable combination of mild spasticity, visual field defects, learning disabilities and headaches.	[17]
13	c.1507G > A p.(Gly503Arg)	15	hom	n.s.	42y	infertility	[8]
14	c.709G > A p.(Trp570*)	15	hom	44y	n.s.	mild cerebellar ataxia and a variable combination of chorioretinopathy	[17]
15	c.709G > A p.(Trp570*)	15	hom	57y	n.s.	mild cerebellar ataxia and a variable combination of chorioretinopathy, tinnitus and vertigo	[17]
16	c.1769A > C p.(His590Pro)	16	hom	n.s.	52y	bilateral optic atrophy	[20]
17	c.2257C > T p.(Arg753*)	20	hom	22y	38y	mild intention tremor, speech impairment, mild memory decline, tinnitus in both ears, and dizziness.	[12]
18	Deletion: (chr3:184070523–184072763) x0 GRCh37/hg19	13–19	hom	20y	30′s	extensive subcortical leukoencephalopathy, mild cerebellar ataxia, a postural tremor and pyramidal signs of the four limbs	[21]

This table lists previous reports in the medical literature of patients and families with *CLCN2*-Related Leukoencephalopathy / Leukoencephalopathy with Ataxia. Description of the variants is as provided in the cited literature; some publications have not provided transcript designations or exact chromosomal positions. The human *CLCN2* gene has 4 known transcripts (NCBI RefSeq and GENECODE v38); variant designation refers to canonical NM_004366.5 / ENST00000265593.4. Modified from reference [12]. hom; homozygous; m: months; y: years; n.s.: not stated.

The novel *CLCN2* variant was initially classified as variant of uncertain significance. To determine co-segregation of the novel *CLCN2* variant with disease in the family, it was assessed by Sanger sequencing in all family members (Fig. 1). Both parents and one unaffected sister were heterozygote carriers, but the brother whom we considered unaffected when we briefly met him to take samples for genetic family testing (**II:1**), was homozygous. He provided informed consent for us to access to his medical records, and it turned out that he had undergone assessment in an infertility clinic. A testicular biopsy had shown a cessation of spermatogenesis at the spermatocyte level with no obvious mature sperm cells (Fig. 1 D). Infertility due to azoospermia has previously been described in two male patients with *CLCN2*-related leukoencephalopathy [6], [8], but findings from testicular biopsy has not been reported so that the exact type of the azoospermia has remained unknown. Mice homozygous for a truncating *CLCN2* variant showed severe degradation of spermatogenesis and shortages of spermatocytes, spermoblasts and sperm cells [9]. Subsequent testing revealed that also his brother (II:4) has azoospermia but he has not undergone testicular biopsy.

The clinical and radiological features of II:1, II:2, and II:4 were well compatible with *CLCN2*-related leukoencephalopathy, and the MRI findings are extremely unusual and almost pathognomonic for this disorder. We thus concluded that the novel homozygote *CLCN2* c.607 > T p.(Gly203Cys) variant causes *CLCN2*-related leukoencephalopathy in family members II:1, II:2 and II:4, but that all three also carried homozygous likely pathogenic CYP2U1 variants and that II:2 and II:4 had the full clinical phenotype of SPG56. We have no explanation for the lack of overt SPG56 findings in II:1, but recent work has implicated folate deficiency in SPG56 [7]. Although serum folate levels were normal in all three patients (12–20 nmol/l, laboratory’s lower reference: 6 nmol/l), we cannot exclude possible deficiency in earlier life.

Formal probabilistic reevaluation of *CLCN2* c.607 > T pathogenicity revealed PM2, PM3 (supporting, because of homozygosity), PP1, PP3, PP4, resulting in a classification of “likely pathogenic”, albeit closer to “variant of uncertain significance” [6]. We suggest that for very rare disorders with highly specific clinical (for example, azoospermia) or biomarker (for example, MRI) profiles, a definitive genetic diagnosis can be made also in the absence of functional data or additional families with the same variants, and that current guidelines for the interpretation of genetic variants in human disease [10] are too strict, precluding clinically valuable diagnoses for patients with very rare diseases. Furthermore, we can confirm that male infertility is part of *CLCN2*-related leukoencephalopathy in humans and present the first testicular biopsy that shows that the mechanism behind the azoospermia is incomplete maturation arrest. Our detailed ophthalmological examination showed a phenotype that agrees well with previous descriptions of findings associated with mutations in the *CYP2U1* and *CLCN2* genes [2], [4], [11], [12].

Next generation sequencing (NGS)-based testing can identify the genetic cause in 12.1 of 61.8 % of patients for ataxia/spastic paraplegia [13]. Increased use of such testing in clinical diagnostics will likely increase the number of patients and families with co-existing disorders. A retrospective study has shown that in up to 5 % of patients referred for NGS tests, molecular diagnoses of 2 diseases with distinct or overlapping phenotypes can be made [14]. A large-scale study from Saudi Arabia recently determined that about 3 % of consanguineous families, examined because of a family history for a Mendelian disease, had genetic evidence for more than one recessive disease [15].

A relatively large body of literature has previously described “expansions of the phenotype” of known monogenic disorders. Although a certain amount of phenotypic variability may be observed in genetically defined human disorders, some of the more unusual phenotypes might also be caused by the co-occurrence, in an individual or family, of a second genetic disorder, especially in consanguineous families. Considerable multidisciplinary clinical and genetic workup can be required to prove or refute the pathogenicity of novel variants, and to delineate core phenotypes for genetic disorders, especially for monogenic recessive disorders in children of consanguineous parents.

## CRediT authorship contribution statement

**Wejdan Almasoudi:** Conceptualization, Data curation, Investigation, Methodology, Project administration, Writing – original draft. **Christer Nilsson:** Investigation, Methodology, Writing – review & editing. **Ulrika Kjellström:** Investigation, Methodology, Visualization, Writing – review & editing. **Kevin Sandeman:** Investigation, Methodology, Visualization, Writing – review & editing. **Andreas Puschmann:** Conceptualization, Funding acquisition, Methodology, Supervision, Visualization, Writing – review & editing.

## Declaration of Competing Interest

The authors declare that they have no known competing financial interests or personal relationships that could have appeared to influence the work reported in this paper.
